# Reduced body length and morphological disorders in *Chrysomya albiceps* (Diptera: Calliphoridae) larvae reared on aluminum phosphide-treated rabbits

**DOI:** 10.1038/s41598-022-12351-w

**Published:** 2022-05-19

**Authors:** Saeed El-Ashram, Noura A. Toto, Abeer El Wakil, Maria Augustyniak, Lamia M. El-Samad

**Affiliations:** 1 Faculty of Science, Kafrelsheikh University, Kafr El-Sheikh, 33516 Egypt; 2 Department of Zoology, Faulty of Science, Damanhour University, El Beheira, Egypt; 3 Department of Biological and Geological Sciences, Faculty of Education, Alexandria University, Alexandria, Egypt; 4 Institute of Biology, Biotechnology and Environmental Protection, Faculty of Natural Sciences, University of Silesia in Katowice, Bankowa 9, 40-007 Katowice, Poland; 5 Department of Zoology, Faulty of Science, Alexandria University, Alexandria, Egypt

**Keywords:** Zoology, Medical research

## Abstract

Assessing the time of death based on the growth and development of insects is a critical task in forensic entomology. The rate of larvae development can be affected by a variety of toxins, including pesticides. Aluminum phosphide (AlP) is a low-cost insecticide that has yet to be tested for entomotoxicological significance, despite the fact that it is frequently the cause of fatal poisoning. In this study, we measured the body length of *Chrysomya albiceps* larvae reared on the carcasses of rabbits poisoned with AlP and analyzed the morphological changes of the larvae reared on the carcasses of rabbits poisoned with AlP. The concentration of AlP in the body of the larvae was significantly lower than in rabbit tissues. Insects from the AlP group had a significantly lower gain in body length. Furthermore, deformities in the larvae were found. Smaller respiratory spiracles were found, as well as a deformed small posterior end with hypogenesis of the posterior respiratory spiracles. Thus, disturbed growth and development of carrion flies found at a crime scene could indicate pesticide poisoning, such as aluminum phosphide.

## Introduction

Insects and other arthropods can be found in virtually every ecological niche. As a result, it's not surprising that they're found at crime scenes. Insects play an important role in tissue decomposition, so many species have become useful in medicolegal forensic entomology^1^, which studies and uses data about insects and their development to help solve criminal cases. Various entomological pieces of evidence, such as eggs, larvae at various stages, pupae, imagoes, or indirect traces of insect existence (e.g., exuviae, feces), appear on a corpse over time after death. A careful examination of collected material, patterns of entomological succession, and the rate of development of insects can assist in determining the time of death. However, in interpreting the data a great precision and caution are required as the obtained results may be expressed/interpreted as postmortem interval (PMI), minimum postmortem interval (mPMI), postcolonization interval (PCI), time of colonization (TOC), or period of infestation (POI)^2,3^. Using the colonization time to estimate PMI or mPMI requires considering many possible assumptions, such as unrestricted and immediate access of insects to the body, known temperature, the certainty that collected material represents the initial colonizers, and others. For this reason, Tarone and Sanford^4^ propose, when using death interval terms, to present them as a null hypothesis with an appropriate list of assumptions. Thus, forensic science is beneficial in estimating the time since death, and the location and cause of death, particularly when poisoning or drug overdose are suspected^5^. It helps to assist in death investigation by limiting the investigation period and thus narrowing down the number of suspects in homicide cases. It also provides valuable information in establishing the timeline of events that lead to death.

Entomotoxicology is focused on analyzing entomological evidence to determine whether or not toxins, drugs, e.g., opiates, were used before death. Notably, the development rate of carrion-feeding insects and toxins' concentrations in their body are evaluated during an investigation, especially when tissue analysis is difficult or even impossible^6^. Therefore, the entomotoxicology and entomological evidence are immensely useful as alternative matrices when the corpse is highly decayed or even skeletonized^7,8^. It is critical to keep in mind; however, that drugs or toxins (as well as insecticides) found in decomposing remains can affect the developmental cycle of insects. Direct ingestion of toxins by insects via tissue or transmission through the food chains of necrophagous/predatory insects can eventually impair the accuracy of PMI (or mPMI) estimation^9–11^. This is why it is necessary to have a thorough understanding of the relationship between the rate of development of a particular insect species and the concentration of a toxic substance in the insects' body and corpse tissues. A morphological examination of the larvae is equally valuable. Flies are insects of the order Diptera^3^. The Calliphoridae, Sarcophagidae, and Muscidae families are the most visible necrophagous flies that specifically degrade organic matter from carcasses of many types. Because of their high sensitivity to chemical signals released during the decomposition process, they appear immediately after death^4,5^. Blow-flies are the most common flies found on remains (Diptera: Calliphoridae). As a result, Calliphoridae larvae, particularly flies found in corpses and/or at death scenes, are frequently used as entomological evidence in forensic investigations, estimating time of death and determining toxic substances^6,7^. *Chrysomya albiceps* is one of the most important forensic blowflies. *C. albiceps* larvae are also known to infest living humans and animals, causing myiasis^8^. *C. albiceps* flies appear early on human corpses and reproduce quickly^18^. Furthermore, their larvae are thought to be the most significant consumers of decomposing tissues^6^. Aluminum phosphide (AlP), also known as "rice tablet," is a low-cost pesticide that is used as an insecticide, rodenticide, and fumigant for crop protection during storage and transportation^9^. Human poisoning with AlP as a result of suicide attempts or accidental environmental exposure (e.g., during fumigation) is associated with a high rate of mortality, owing in part to the development of severe metabolic acidosis^10^. When AlP comes into contact with acid (gastric acid) or moisture, phosphine (PH3), a toxic gas, is released. Acute aluminum phosphide poisoning primarily affects the heart, lungs, and liver. Unfortunately, the high toxicity of AIP and the lack of a specific antidote increase the risk of death from AIP poisoning in humans^11,12^. Cardiovascular toxicity is the leading cause of death^13^.

This study aimed to assess the effect of AlP on the growth rate, morphology, and AlP concentration in *Chrysomya albiceps* larvae, grown on the tissues of AlP-treated rabbits. For this purpose, the flies' body length gain was evaluated up to102 hours of insects' life. The content of AlP in rabbit tissues and the 3rd larval stage of *C. albiceps* were estimated by high-performance liquid chromatography (HPLC). Furthermore, a scanning electron microscope analysis of the larval morphology allows us to describe AlP-induced deformations in *C. albiceps* larvae.

## Materials and methods

### Ethical statement

The ethics committee at Alexandria University approved all animal-related laboratory procedures. All animal experiments were carried out in compliance with Alexandria University's Guidelines regarding the Use and Care of Experimental Animals (Alex-Univ 72020). All procedures were recorded in compliance with ARRIVE standards.

### Experimental design

Six adult male rabbits, weighing on average 1.25 kg, were used to rear one generation of insects that were attracted to the rabbit carcasses and invaded them. The rabbits were acclimated in iron cages at Alexandria University's Faculty of Science for two weeks at 30 ± 4 °C, under a light–dark cycle (12:12 h), with unlimited access to food and water. Following acclimation, rabbits were randomly assigned to one of two groups: the control group received sterile water intragastrically twice daily via a gastric tube for one week; or the experimental group received 27.4 mg AlP/kg body weight via a gastric tube twice daily until each animal was sacrificed. Concentrations of lethal doses were determined as previously described^13^. Before procedure of intoxication, animals were slightly sedated by an intramuscular injection of Xylazine (5 mg/kg). Finally, rabbits were euthanized by overdosage of Isoflurane (> 4.5% in oxygen flow at about 2L/min) until respiratory arrest occurred for more than 1 min. The carcasses were placed in a terrestrial environment during the experiment. Blood samples were collected from the retro-orbital sinus of each control and treated rabbit 12 h after the initial dose administration. According to the protocol used at Alexandria University, the rabbits were sacrificed at the end of the study period. After the animals died, liver, kidney, fat, lung, and muscle samples (1 g of each tissue) were collected. Thirty adult *C. albiceps* flies were collected and identified using current keys (20 females and 10 males)^14^. Flies were transferred to cages after identification and kept under controlled conditions with an average temperature of 30 ± 4 °C, a light–dark cycle of 12:12 h, and a relative humidity of 60 ± 10%. The cages were protected with an external net curtain to avoid other insect species' invasion^15^. The adult flies were reared on the cadavers of rabbits that were divided into control and treated groups. The hatching of eggs was checked every three hours. Larvae, pupae, and adults were checked every twelve hours until adults' emergence^16^.

### Morphometric studies

The length (mm) of *C. albiceps* larvae was determined using a Vernier Caliper at various time intervals (12, 24, 36, 48, 60, 72, 84, and 102 h).

### A sampling of larvae for quantitative analysis of AlP

Twenty third instar larvae were randomly collected five days after egg hatching from all carcasses of rabbits in the control and treated groups and then rinsed in phosphate buffer, frozen, and stored at − 80 °C until toxicological analysis^17^.

### Sample analysis and HPLC conditions

HPLC (Agilent 1260) with a UV–Visible spectrophotometric detector was used to determine the concentrations of AlP in blood, liver, kidney, muscles, fats, lung, and larvae from both control and treated groups. Table 1 showed the optimized chromatographic conditions used to separate the samples.

**Table 1:** Optimized chromatographic conditions.

Parameter	Optimized condition
Chromatograph	HPLC (Agilent 1260)
Column	Eclipse Plus C18 (4.6 mm × 100 mm)
Flow rate	1 mL/min
Detection wavelength	218 nm
Mobile phase	Trifluoroacetic acid in water, acetonitrile, and methanol (70:25:5 v/v)
Injection volume	20 μl
Column temperature	40 °C

### Scanning electron microscopy (SEM)

The third instar larvae from the control and treated groups were collected, prepared, and scanned by a scanning microscope JEOL (JSM-5300) at the Electron Microscope Unit (EMU), Faculty of Science, Alexandria University, Egypt.

### Statistical analysis

TIBCO Statistica 13.3.0 (https://docs.tibco.com/products/tibco-statistica-13-3-0) was used to process the data. Regression equations and correlation coefficients were calculated to compare the body length gain of both larvae groups. A comparison of the slopes of regression lines procedure was used to compare the two groups. The concentration of AlP in the control group's rabbit tissues was below the detection limit. ANOVA (LSD test, *p* < 0.05) was used to compare the AlP concentration in rabbit tissues from the treated group and in larvae.

## Results

**Figure 1:**
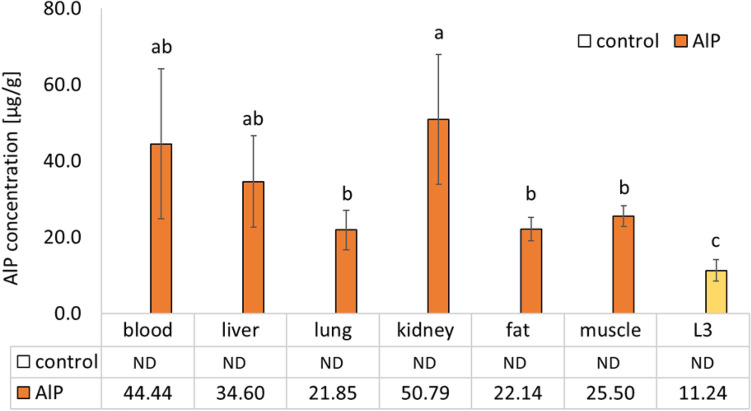
Aluminum phosphide (AlP) concentrations (mean ± SD) in blood and tissues of AlP-treated rabbits (AlP group) and the third instar of *Chrysomya albiceps* larvae (L3) fed on rabbits' carcasses. The concentration of AlP in the control group was below the detection limit. The same letters denote no significant differences (ANOVA, LSD test, *p* < 0.05). *ND* not detect.

Aluminum phosphide was not found in the control group's rabbit tissue or the third larval instar's body. The highest concentrations of this substance were found in the kidney, blood, and liver of rabbits in the AlP-treated group, at 50.79 μg/g, 44.44 μg/mL, and 34.60 μg/g, respectively. These mean values were not significantly different from each other. Whereas, significantly lower concentrations of AlP were found in muscles (25.50 μg/g), fat (22.14 μg/g), and lungs (21.85 μg/g) comparing with the value measured in kidney and blood. The concentration of AlP in the third larval instar of *C.* *albiceps* was 11.24 μg/g, and the value was significantly lower than in the rabbits' tissue (Fig. 1).

**Figure 2:**
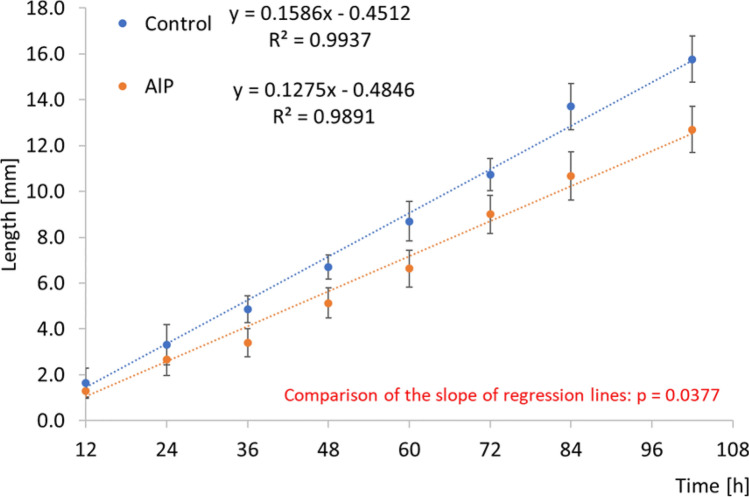
Length of *Chrysomya albiceps* larvae (mean ± SD) at different time points. Larvae were fed on AlP-treated rabbits' carcasses (the AlP group) or sterile water (the control group) before death. Dotted lines mark regression lines. The regression line's parallelism was tested by comparing the regression lines' slope (GLM, equal slopes model).

The larvae of both experimental groups showed a regular body length gain over the 108 h of the experiment, although their development was slowed down in the AlP group (Fig. 2).

**Figure 3:**
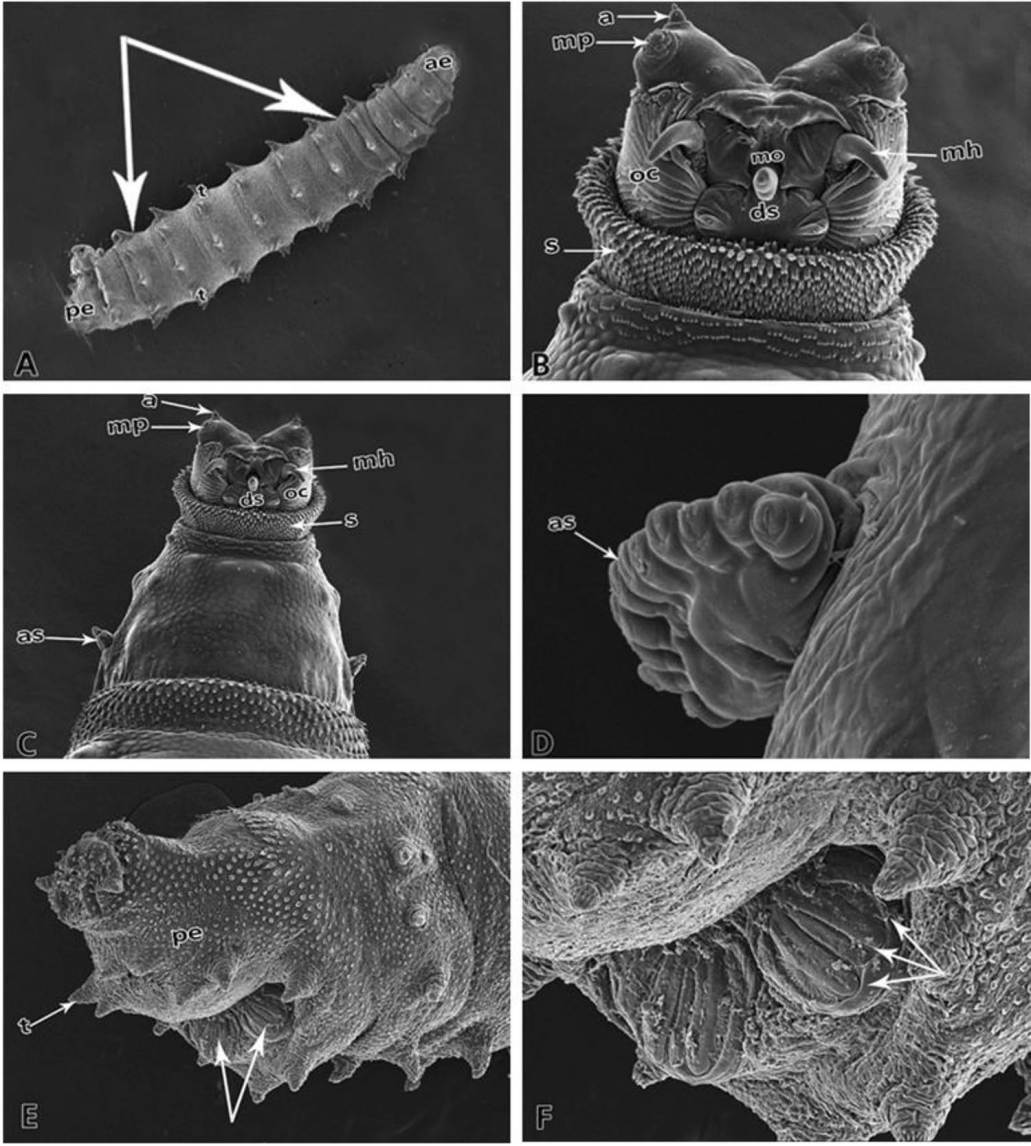
Scanning electron micrographs of a third instar larva of *Chrysomya albiceps*. (**A**) Larval body composed of groups of tubercles (t) located at the anterior and posterior ends of each segment, anterior (ae), and posterior ends (pe), abdominal segments (arrows). (**B**) Cephalic region with antennae (a), maxillary palp (mp), spines (s), dental sclerite (ds), mouth hooks (mh), oral cristae (oc), and anterior spiracle (as). (**C**) Details of antennae (a) and maxillary palp with five papillae and anterior spiracle (as), spines (s), oral cristae (oc), and dental sclerite (ds). (**D**) Details of anterior spiracles (as) in a row. (**E**) Anal segment with posterior spiracles (arrows). (**F**) Details of anal segment with three spiracular openings (arrows).

The control group larvae achieved a final average length of 15.77 mm, while the AlP group larvae only 12.70 mm. The slope regression lines' comparison revealed a significant difference in the slope of both lines (*p* = 0.0377), confirming significant differences in the larvae's growth rate in both groups. Ultrastructural examination of *C. albiceps* third instar larvae from the control groups showed regular larval bodies with a normal appearance of the posterior end (Fig. 3A). The control larvae demonstrated a standard appearance of the anterior end with regular hooks (Fig. 3B) and normal anterior respiratory spiracles (Fig. 3C,D). The control larvae's posterior end demonstrated normal processes and normal posterior respiratory spiracles (Fig. 3E,F).

**Figure 4:**
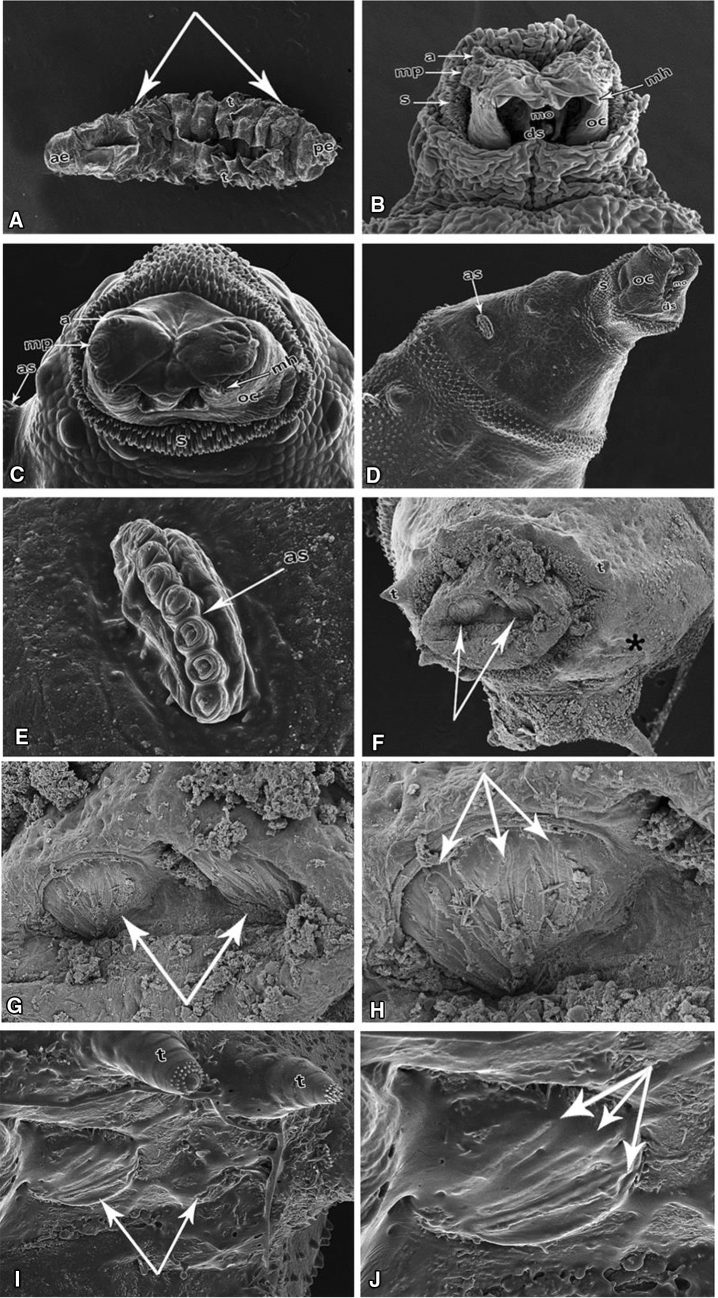
Scanning electron micrographs of a third instar larva of *Chrysomya albicep*s from the AlP group. (**A**) Larval body composed of groups of tubercles (t) located at the anterior and posterior ends of each segment, anterior (ae), and posterior ends (pe). Notes: Shrinkage in larval length, arrows abdominal segments. (**B**) Ventral view of the cephalic region with antennae (a), maxillary palp (mp), spines (s), dental sclerite (ds), short mouth hooks (mh), oral cristae (oc). (**C**) Dorsal view of the cephalic region with antennae (a), maxillary palp (mp), spines (s), dental sclerite (ds), short mouth hooks (mh), oral cristae (oc), and deformed anterior spiracle (as). (**D**) Details of antennae (a) and maxillary palp with five papillae and deformed anterior spiracle (as), spines (s), oral cristae (oc), and dental sclerite (ds). (**E**) Details of anterior spiracles (as) in a row. (**F**) Anal segment with posterior spiracles (arrows), tubercles (t). (**G**) Magnified part of the micrograph. (**F**) Details of posterior spiracles. (**H**) Details of the anal segment with deformed three spiracular openings (arrows). (**I**) Hypogenesis of posterior spiracles (arrows). (**J**) Completely deformed three spiracular openings (arrows).

Ultrastructural examination of *C. albiceps* third instar larvae reared on AlP-treated rabbits demonstrated dense, compressed shape arched body (Fig. 4A) and deformed anterior end with much smaller mouth hooks (Fig. 4B), deformed small-sized anterior respiratory spiracles (Fig. 4C–E).

The posterior respiratory spiracles revealed hypogenesis compared to the control larvae (Fig. 4F,G–J).

## Discussion

Insects feeding on corpse tissues absorb the same substances as in a deceased body before death. Many compounds that cause fatal poisoning can be identified in insects' bodies found in the remains^18^. Numerous studies have demonstrated the effectiveness of chemical detection, identification, and quantification in the bodies of insects such as flies, confirming their critical role in poisoning research and utility in criminal investigations^7^. Drugs can be detected in maggots, which can provide useful information about the type and concentration of drugs in a deceased body. As a result, in some cases, it is possible to identify the primary food source/tissue on which the larvae rely^19^. In our research, we found different AlP concentrations in particular rabbit tissues. The highest concentrations were found in the kidney and liver (Fig. 1), which is reasonable given these organs' function in detoxification and eliminating toxins. In the lungs, muscles, and fat, the concentration of AlP was lower. Similar results were obtained by El-Samad et al.^20^ who detected tramadol by HPLC in various rabbits' organs experimentally injected with this substance. Importantly, AlP concentration in rabbit tissues was significantly higher than in the body of the third instar of *C. albiceps*. Data on the relationship between the concentration of toxins in tissues of deceased and insects are, to some extent, divergent. Some scientists found a direct correlation between the toxin/drug concentration in larvae and cadaver tissues. Still, others found no correlation or postulated that xenobiotic concentrations found in larvae could be significantly lower than in body tissues^18^. For example, Introna et al.^21^ found that morphine concentrations in *C. vicina* larvae feeding on decomposing liver tissues of deceased humans who died of morphine poisoning were highly correlated with post-mortem tissue concentrations. On the contrary, Nolte et al.^22^ revealed that concentrations of cocaine in larvae were significantly lower than those observed in tissues. More recent studies provide complementary information. Campobasso et al.^23^ investigated the correlation between xenobiotic concentrations in the human liver and larvae of the Calliphoridae family (*Lucilia sericata*) feeding on the tissue. Opiates, cocaine, barbiturates, and antidepressants were among the toxins tested. All of the xenobiotics found in *L. sericata* bodies were in lower concentrations than in human tissues. Thus, the concentration of many substances in insect tissues is often lower than in the source. This is because many toxins are partly metabolized and gradually excreted by insects. Undoubtedly, one must agree that the larvae can have relevance in the qualitative identification of a specific substance. Kintz et al.^24^ reported that morphine and phenobarbital were detected in Calliphoridae larvae developed on cadavers of chronic heroin abusers two months after their death. However, quantitative evaluation, estimated based on the correlation between the toxin concentration in larvae and tissues, should be interpreted with great caution^18,23^.

Toxins also affect the growth rate of fly larvae developing on the corpses. Many studies have assessed the impact of drugs/pesticides on the morphometry of fly species^25,26^ and their development^20,27,28^ in terms of forensic entomotoxicology. The vast majority of drugs accelerate the development of larvae, while pesticides tend to delay it. Larval development can be accelerated by diazepam^14^, codeine^29,30^, heroin^31^, methamphetamine and its metabolite, p-hydroxymethamphetamine^32^. However, hydrocortisone and a barbiturate—sodium methohexital^33^ and the insecticide DEET—N, N-Diethyl-meta-toluamide^34^ can delay the development of fly larvae. Ketamine has no significant effect on the larvae's length and mass^35^. Data on the effects of tramadol on the development of fly larvae are additionally somewhat divergent. Elshehaby et al.^36^ found that tramadol retards larval development of *C. albiceps*. Also, El-Samad et al.^20^ demonstrated that *Lucilia sericata* larvae reared on rabbits administered tramadol had a prolonged developmental period. Whereas Ekrakene and Odo^37^ established that the larvae from the tramadol reared group gained body length and weight better than the control groups. Abou Zied^38^ also reported that *Sarcophaga argyrostoma* larvae reared on rat carcasses containing tramadol had significantly longer total body length than the control larvae. Also, morphine can accelerate or delay insect larval development, depending on the studied species^5,39,40^. In our study, we found that AlP negatively affects the length of *C. albiceps* larvae (Fig. 2), which seems to be logical, taking into account that AlP is also used as an insecticide. This information should be considered when estimating the time of death. In our experiment, insects had free and immediate access to the body, and collected insects represented the initial colonizers. However, AlP limited the growth of the larvae, and therefore the estimated time of death (when AlP is concerned) should be perceived as the minimum time of death (mPMI).

In the present study, the examination of *C. albiceps* by SEM revealed that third instar larvae reared on AlP intoxicated rabbits demonstrated a dense compressed body with a deformed appearance of the anterior end, small oral hooks, and deformed small-sized spiracles. The posterior end was distorted with deformed posterior respiratory spiracles. Our results confirm the view of Elshehaby et al.^36^, who showed that tramadol caused abnormal fused small-sized respiratory spiracles and deformed small posterior end with hypogenesis of the posterior respiratory spiracles in the third instar larvae of *C. albiceps*. Also, the opiate drug (codeine) was reported to cause morphological changes in *C. albiceps* larvae. These changes were in the form of deformed body segments, abnormalities in the shape of anterior and posterior spiracles. However, the same study reported that codeine accelerates the development rate during the life cycle of *C. albiceps*^29^. Smaller mouth hooks can directly or indirectly affect the rate and amount of food consumed. This AlP-induced anatomical change could thus indirectly contribute to a reduction in the growth rate. However, an in-depth research is needed to confirm this assumption. Nevertheless, it can be presumed that the larval deformation can indicate the deceased's poisoning with insecticides, e.g., aluminum phosphide.

## Conclusions

Aluminum phosphide caused delay of growth and deformation of the larvae of *C. albiceps* with small-sized respiratory spiracles and deformed small posterior end with hypogenesis of the posterior respiratory spiracles. Results can be helpful to establish evidence while determining the cause of death. Analysis of the duration of the life cycle can aid in estimating the time of death. The evidence held by insects in a crime scene is not prone to destruction.
